# Impact of waiting time after surgery and overall time of postoperative radiochemotherapy on treatment outcome in glioblastoma multiforme

**DOI:** 10.1186/s13014-015-0478-5

**Published:** 2015-08-16

**Authors:** Annekatrin Seidlitz, Timo Siepmann, Steffen Löck, Tareq Juratli, Michael Baumann, Mechthild Krause

**Affiliations:** Department of Radiation Oncology, Faculty of Medicine and University Hospital Carl Gustav Carus, Technische Universität Dresden, Fetscherstr. 74, 01307 Dresden, Germany; OncoRay-National Center for Radiation Research in Oncology, Faculty of Medicine and University Hospital Carl Gustav Carus, Technische Universität Dresden, Helmholtz-Zentrum Dresden-Rossendorf, Dresden, Germany; German Cancer Consortium (DKTK), Dresden and German Cancer Research Center (DKFZ), Heidelberg, Germany; Center for Clinical Research and Management Education, Division of Health Care Sciences, Dresden International University, Dresden, Germany; Department of Neurology and Department of Psychotherapy and Psychosomatic Medicine, University Hospital Carl Gustav Carus, Technische Universität Dresden, Dresden, Germany; Department of Neurosurgery, University Hospital Carl Gustav Carus, Technische Universität Dresden, Dresden, Germany; Helmholtz-Zentrum Dresden–Rossendorf, Institute of Radiooncology, Dresden, Germany

## Abstract

**Background:**

A time factor of radiooncological treatment has been demonstrated for several tumours, most prominently for head and neck squamous cell carcinoma and lung cancer. In glioblastoma multiforme studies of the impact of postoperative waiting times before initiation of radio- or radiochemotherapy were inconclusive. Moreover analysis of the impact of overall treatment time of radiochemotherapy as well as overall duration of local treatment from surgery to the end of radiochemotherapy is lacking to date.

**Methods:**

In this retrospective cohort study, we included 369 consecutive patients treated at our institution between 2001 and 2014. Inclusion criteria were histologically proven glioblastoma multiforme, age ≥ 18 years, ECOG performance status 0–2 before radiotherapy, radiotherapy or radiochemotherapy with 33 × 1.8 Gy to 59.4 Gy or with 30 × 2.0 Gy to 60 Gy. The impact of postoperative waiting time, radiation treatment time and overall duration of local treatment from surgery to the end of radiotherapy on overall (OS) and progression-free (PFS) survival were evaluated under consideration of known prognostic factors by univariate Log-rank tests and multivariate Cox-regression analysis.

**Results:**

The majority of patients had received simultaneous and further adjuvant chemotherapy, mainly with temozolomide. Median survival time and 2-year OS were 18.0 months and 38.9 % after radiochemotherapy compared to 12.7 months and 12.6 % after radiotherapy alone. Median progression-free survival time was 7.5 months and PFS at 2 years was 14.3 % compared to 6.0 months and 3.3 %, respectively. Significant prognostic factors in multivariate analysis were age, resection status and application of simultaneous chemotherapy. No effect of the interval between surgery and adjuvant radiotherapy (median 27, range 11–112 days), radiation treatment time (median 45, range 40–71 days) and of overall time from surgery until the end of radiotherapy (median 54, range 71–154 days) on overall and progression-free survival was evident.

**Conclusion:**

Our data do not indicate a relevant time factor in the treatment of glioblastoma multiforme in a large contemporary single-centre cohort. Although this study was limited by its retrospective nature, its results indicate that short delays of postoperative radiochemotherapy, e.g. for screening of a patient for a clinical trial, may be uncritical.

## Background

Glioblastoma multiforme (GBM) is the most common and malignant primary brain tumour. Current standard therapy consists of radical surgical resection followed by radiotherapy combined with concomitant and adjuvant chemotherapy using temozolomide [1]. Overall prognosis of GBM remains dismal, although different prognostic groups may be distinguished, e.g. by the commonly used recursive partitioning analysis (RPA) classification which was worked out by the Radiation Therapy Oncology Group (RTOG) consortium [2–5].

In many tumours, a negative impact of delayed radiotherapy or prolonged treatment time has been demonstrated, most prominently for head and neck squamous cell carcinoma and lung cancer, but also for slowly proliferating tumours such as breast and prostate cancer (e.g. [6–8], reviewed in [9–11]). The most likely mechanism is repopulation before and/or during treatment [10, 12].

Presence of a time factor in aggressively proliferating GBM may be expected, but has not been systematically evaluated so far. This research topic is relevant as optimal schedules of radiation for GBM are still an open question. In addition, because of the poor prognosis, patients with GBM should be encouraged to be treated within clinical trials testing new treatment strategies. These trials require intensive, e.g. molecular predictive methods, which in many cases may delay start of radiochemotherapy due to the screening procedures. However, evidence that the time factor could be one reason for the poor outcome of GBM despite intense therapy is sparse. Whereas results on the impact of postoperative waiting times before initiation of radio- or radiochemotherapy are inconclusive [13], evidence of the impact of overall treatment time of radiochemotherapy is lacking.

Therefore, the aim of this retrospective analysis was to investigate the time factor in a contemporary cohort of glioblastoma patients treated with high dose radiotherapy.

## Patients and methods

### Patients and inclusion criteria

We included patients who had been treated with radio- or radiochemotherapy for newly diagnosed GBM between November 2001 and January 2014 at the Department of Radiation Oncology, University Hospital Carl Gustav Carus, Dresden. The patients were treated according to the current standard at the particular time with the majority according to interdisciplinary tumour board recommendations under consideration of individual patient factors. Inclusion criteria for time factor evaluation were: age ≥ 18 years at start of radio (chemo) therapy (RCT), performance status better or equal Eastern Cooperative Oncology Group (ECOG) performance status 2 (complying with Karnofsky performance status ≥ 50), histologically proven glioblastoma multiforme or imaging typical for GBM in case of borderline histology results and applied radiation dose of 59.4 Gy (1.8 Gy/fraction) or 60 Gy (2 Gy/fraction) with or without simultaneous and adjuvant chemotherapy. Patients were excluded after previous cranial irradiation and if the prescribed dose was not applied. We included a total of 369 consecutive patients. This retrospective study was approved by our local institutional review board (IRB number: EK 18012014).

### Radiooncological treatment and follow up

**Fig. 1:**
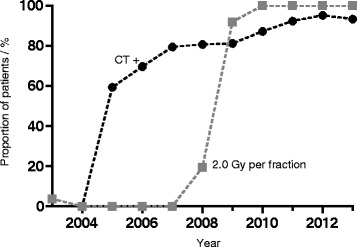
Diagram showing changes of treatment over time. Proportions of patients with and without chemotherapy (CT) as well as proportions of different fractionation schedules are shown

Radiotherapy fractionation changed from 33 × 1.8 to 30 × 2.0 Gy per fraction resulting in total doses changing from 59.4 Gy to 60 Gy during the study period (Fig. 1). Total dose was prescribed to a clinical target volume including the surgical cavity and contrast-enhancing lesions visible in post-operative MRI, extended by a 20 mm margin for the majority of patients according to the trial-26981-22981 by the European Organisation for Research and Treatment of Cancer. Only 7 patients were treated slightly different according to the RTOG guidelines within a prospective clinical trial requiring shrinking field technique so that the boost of 10 Gy was applied to a smaller volume with reduced margins of 5 mm. After 2008, co-registration of post-operative MRI with radiation planning CT was possible and used for treatment planning. Radiotherapy was delivered with linear accelerators providing photons of energies ≥ 6MV. The field shaping device could be blocks or multileaf collimator. 3D-conformal radiotherapy plans were generated within a dedicated planning system. IMRT was used if appropriate with respect to target volume coverage or normal tissue sparing.

Two hundred forty eight patients (67.2 %) received simultaneous chemotherapy and were prescribed further adjuvant chemotherapy (Fig. 1), mainly with temozolomide in standard dosage (simultaneous: 75 mg/m^2^ daily during radiotherapy; adjuvant: 6 cycles of 150–200 mg/m^2^ for 5 days during each 28-day cycle); however before 2005, some patients also received other alkylating agents like nimustin. Very few patients were treated with additional drugs within clinical trials, e.g. the angiogenesis inhibitor bevacizumab or the integrin inhibitor cilengitide. Only in one of these trials, temozolomide was replaced by another drug (bevacizumab and irinotecan: Glarius trial 2009-010390-21). The trials which had already been analysed and published were negative, i.e. the additional drugs had no impact on survival in the particular studies [14, 15]. Patients underwent pre-and postoperative MRI within 48 h if they had no contraindications. After biopsy only, no post-operative MRI was performed. Follow-up MRI was usually performed every 3 months. Neurosurgical resection preceding adjuvant therapy included biopsy, gross total or partial resection. The last two in that series were recorded according to operative report and validated by contrast enhancement in early postoperative imaging.

### Endpoints

Endpoints were overall survival (OS) and progression-free survival (PFS). They were calculated from the date of primary resection until the day of death for OS and until the diagnosis of progression based on imaging or until tumour-related death for PFS, respectively. Non-event data were censored at last follow-up for OS and last imaging investigation without signs of progression for PFS. The endpoints were estimated by the Kaplan-Meier method. The impact of the following time intervals on outcome was evaluated under consideration of known prognostic factors by univariate and multivariate Cox-regression analyses: time interval between resection and start of RCT (TI); radiation treatment time (RTT), i.e. the time from the first to the last radiation fraction; and the overall duration of local treatment (ODT), i.e. the time from resection to the last radiotherapy fraction. Time intervals and age were analysed as continuous variables. The obtained results were verified by an alternative approach, splitting the cohort by the median of the considered factor and performing Log-Rank tests. P values smaller than 0.05 were considered significant. Statistical analysis was performed using the software programs IBM SPSS Statistics 21, STATA 11 (StataCorp LP, College Station, TX, USA) and GraphPad Prism 5.

## Results and discussion

### Patient characteristics and outcome of therapy

All patients treated between 2001 and 2014 with high-dose (59.4-60 Gy) postoperative radiotherapy or radiochemotherapy for newly diagnosed GBM were screened. Patient characteristics of the final cohort comprising 369 patients are shown in Table 1.

**Table 1:** Baseline patient characteristics

	All patients	
Characteristics	Number	Percentage
Age at diagnosis, median (range) [years]	62 (23–86)	
ECOG-status before radiotherapy		
0 (KPS 90–100)	55	14.9
1 (KPS 70–90)	230	62.3
2 (KPS < 70)	84	22.8
RPA class before radiotherapy		
III	21	5.7
IV	192	52.0
V	152	41.2
Unknown	4	1.1
MGMT promoter status		
Unmethylated	30	8.1
Methylated	23	6.2
Unknown	316	85.6
Extent of resection		
Gross total resection	176	47.7
Partial resection	155	42.0
Biopsy only	24	6.5
Undeterminable	14	3.8
Simultaneous chemotherapy	248	67.2
Fractionation		
33 × 1.8 Gy ∑ 59.4 Gy	187	50.7
30 × 2.0 Gy ∑ 60.0 Gy	182	49.3

Abbreviations: KPS-Karnofsky performance status scale. ECOG-Eastern Cooperative Oncology Group

**Fig. 2:**
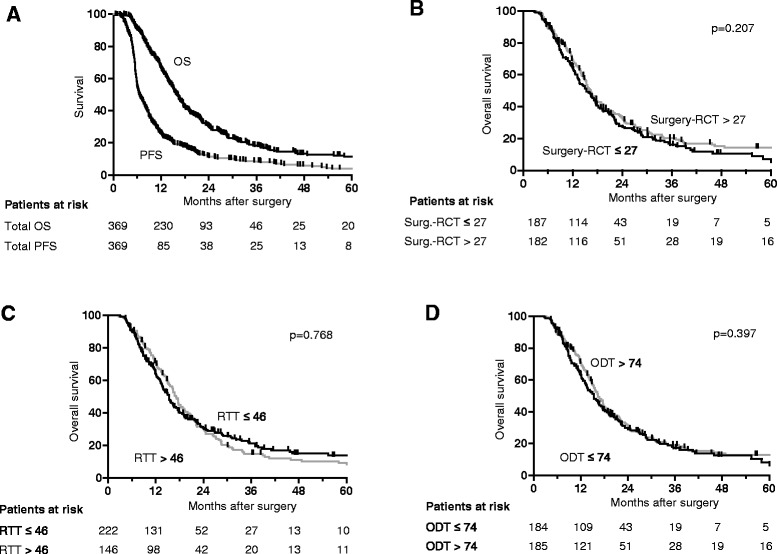
Kaplan-Meier curves showing survival. Overall survival (OS) and progression free survival (PFS) in the whole cohort (**a**) and OS depending on time interval from surgery until initiation of radio- or radiochemotherapy (RCT) (**b**), on radiation treatment time (RTT) (**c**) and overall duration of local treatment from surgery to the end of radio- or radiochemotherapy (ODT) (**d**) are shown

At a median follow-up of 14.9 months (range 4.6-71 months), median survival was 18.0 months with a 2-year overall survival rate (OSR) of 38.9 % after radiochemotherapy compared to 12.7 months and 12.6 % after radiotherapy alone. Progression-free survival was 7.5 months and the 2-y OSR 14.3 % compared to 6.0 months and 3.3 %, respectively (Fig. 2a). The results for patients treated with radiochemotherapy but not for patients treated with radiotherapy alone are slightly better compared to those reported in the EORTC-NCIC landmark study with a median survival of 14.6 months and a 2y-OSR of 26.5 % after radiochemotherapy compared to 12.1 months and 10.4 % after radiotherapy [1, 16]. This is despite the fact that patient age is a prognostic factor and the trial by Stupp et al. was limited to patients ≤ 70 years, whereas our analysis does not include an upper limit of age, leading to a higher median age in our cohort. The better outcome in our radiochemotherapy cohort may be partially explained by the exclusion of patients who did not receive the prescribed radiation dose of 59.4 or 60 Gy in the present study in order to generate a homogeneous cohort for the evaluation of the time factor. The results of Stupp et al. were evaluated by an intent-to-treat analysis as usual for phase III trials. In 5 % of the patients radiochemotherapy was stopped early with an application of <90 % of the prescribed dose. Furthermore our analysis is monocentric, thereby reducing the well-recognized impact of heterogeneous staging procedures, prescription and treatments by different centres. In addition, our centre performs rigorous follow-ups and offers a significant proportion of patients with progression advanced therapy including repeated surgery, reirradiation and further chemotherapy. This is not expected to affect PFS but could affect overall survival and therefore potentially decrease the impact of time intervals on OS.

### Prognostic parameters

**Fig. 3:**
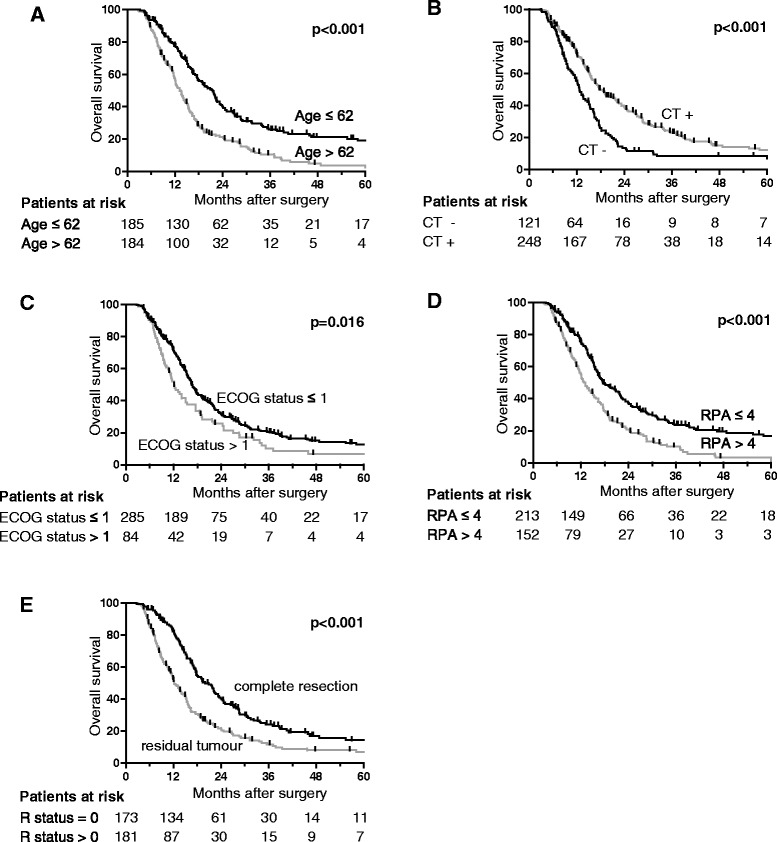
Kaplan-Meier curves showing overall survival depending on prognostic factors. Overall survival is shown depending on age (**a**), simultaneous chemotherapy (CT, **b**), ECOG performance status (**c**), RPA-class (**d**) and resection status (**e**)

Univariate analysis of prognostic and treatment related parameters revealed a significant impact on outcome for age, performance status, RPA-class, O6-methylguanin-DNA-methyltransferase (MGMT) promoter methylation, simultaneous chemotherapy and extent of resection (Table 2, Fig. 3). In multivariate analysis, the parameters age, simultaneous chemotherapy and extent of resection remained significant whereas the independent impact of performance status and RPA-class could not be confirmed (Tables 3 and 4). As analysis of the MGMT methylation status became standard only in the recent years, this parameter was not available for the majority of patients evaluated here and therefore not included in the multivariate analysis. Another reason which might explain the missing independent impact of RPA-class on the results is that age, which is the most powerful RPA criterion, sufficiently explains the results in our cohort of patients. However, parameters that emerge from recursive partitioning analysis should be independent of each other [2, 5, 17]. It therefore appears likely that additional explanations contribute to the different results for RPA-class in our study compared to many other studies, including the retrospective and monocentric nature of our study with some missing data and the risk of centre specific selection and prescription bias. As performance status is the second most important factor for RPA-classing after age, varying ECOG-assessment between different observers is not unlikely. Lastly, a consecutive cohort treated in a routine setting may differ from study populations specifically recruited for a clinical trial or a prospective observational protocol for recursive partitioning analysis.

**Table 2:** Univariate analyses of the impact of prognostic factors on overall survival (OS) and progression free survival (PFS)

OS			PFS	
All patients			All patients	
HR (95 % CI)	*p*-value	Factors	HR (95 % CI)	*p*-value
0.991 (0.981-1.001)	0.089	Interval surgery → RCT	0.994 (0.985-1.003)	0.188
1.017 (0.985-1.049)	0.303	Radiation treatment time	1.008 (0.978-1.039)	0.616
0.994 (0.985-1.003)	0.198	Overall duration of treatment	0.995 (0.987-1.004)	0.277
1.037 (1.026-1.048)	<0.001	Age	1.025 (1.016-1.035)	<0.001
1.389 (1.062-1.816)	0.016	ECOG state ≤ 1 vs > 1	1.241 (0.957-1.610)	0.104
1.768 (1.397-2.236)	<0.001	RPA-class ≤ 4 vs > 4	1.650 (1.315-2.070)	<0.001
1.825 (1.440-2.314)	<0.001	Resection status (total vs. subtotal)	1.560 (1.248-1.951)	<0.001
0.604 (0.474-0.769)	<0.001	Simultaneous CT	0.669 (0.528-0.848)	0.001
0.888 (0.703-1.122)	0.321	Fractionation	1.002 (0.806-1.246)	0.984
0.312 (0.120-0.813)	0.017	MGMT methylation	0.447 (0.220-0.909)	0.026

**Table 3:** Multivariate analyses of overall survival

OS	All patients		Fractionation 33 × 1.8 Gy		Fractionation 30 × 2.0 Gy	
Factor	HR (95 % CI)	*p*-value	HR (95 % CI)	*p*-value	HR (95 % CI)	*p*-value
Interval surgery → RCT	0.998 (0.987-1.009)	0.705	0.985 (0.970-1.001)	0.059	1.007 (0.994-1.020)	0.312
Age	1.033 (1.020-1.045)	<0.001	1.031 (1.015-1.047)	<0.001	1.040 (1.019-1.061)	<0.001
ECOG state > 1	0.984 (0.728-1.364)	0.984	0.956 (0.638-1.431)	0.522	0.974 (0.580-1.636)	0.920
RPA-class > 4	1.210 (0.905-1.617)	0.199	1.240 (0.862-1.784)	0.247	1.159 (0.702-1.912)	0.564
Incomplete resection	1.886 (1.481-2.402)	<0.001	1.588 (1.152-2.189)	0.005	2.265 (1.533-3.347)	<0.001
Simultaneous CT	0.573 (0.427-0.767)	<0.001	0.592 (0.419-0.837)	0.003	0.535 (0.308-0.929)	0.026
Fractionation	1.061 (0.807-1.396)	0.672	-	-	-	-
Radiation treatment time	1.025 (0.985-1.068)	0.225	1.013 (0.960-1.070)	0.634	1.029 (0.964-1.098)	0.394
Age	1.034 (1.021-1.047)	<0.001	1.031 (1.015-1.047)	<0.001	1.040 (1.019-1.061)	<0.001
ECOG state > 1	1.006 (0.739-1.371)	0.968	1.027 (0.692-1.525)	0.893	0.947 (0.565-1.586)	0.835
RPA-class > 4	1.222 (0.914-1.634)	0.175	1.244 (0.692-1.790)	0.239	1.157 (0.700-1.913)	0.570
Incomplete resection	1.927 (1.510-2.460)	<0.001	1.642 (1.183-2.278)	0.003	2.301 (1.558-3.399)	<0.001
Simultaneous CT	0.584 (0.437-0.782)	<0.001	0.570 (0.405-1.801)	0.001	0.581 (0.335-1.009)	0.054
Fractionation	1.175 (0.865-1.595)	0.302	-	-	-	-
Overall duration of treatment	1.000 (0.989-1.010)	0.939	0.987 (0.971-1.002)	0.084	1.008 (0.995-1.021)	0.239
Age	1.033 (1.020-1.045)	<0.001	1.030 (1.014-1.046)	<0.001	1.040 (1.019-1.061)	<0.001
ECOG state > 1	1.005 (0.735-1.375)	0.974	0.858 (0.644-1.443)	0.858	0.976 (0.581-1.640)	0.928
RPA-class > 4	1.209 (0.905-1.616)	0.199	1.231 (0.855-1.773)	0.263	1.169 (0.708-1.931)	0.542
Incomplete resection	1.884 (1.479-2.399)	<0.001	1.571 (1.139-2.166)	0.006	2.256 (1.527-3.333)	<0.001
Simultaneous CT	0.568 (0.424-0.759)	<0.001	0.582 (0.413-0.821)	0.002	0.535 (0.308-0.928)	0.026
Fractionation	1.068 (0.805-1.418)	0.648	-	-	-	-

The impact of time interval from surgery until initiation of radio- or radiochemotherapy (RCT), radiation treatment time and overall duration of local treatment from surgery to the end of radio- or radiochemotherapy on overall survival (OS) is shown

**Table 4:** Multivariate analyses of progression free survival

PFS	All patients		Fractionation 33 × 1.8 Gy		Fractionation 30 × 2.0 Gy	
Factor	HR (95 % CI)	*p*-value	HR (95 % CI)	*p*-value	HR (95 % CI)	*p*-value
Interval surgery → RCT	1.000 (0.991-1.010)	0.943	0.999 (0.985-1.014)	0.896	1.000 (0.987-1.014)	0.984
Age	1.021 (1.010-1.032)	<0.001	1.022 (1.007-1.036)	0.003	1.021 (1.005-1.037)	0.009
ECOG state > 1	0.965 (0.712-1.307)	0.817	0.931 (0.618-1.403)	0.733	0.987 (0.617-1.581)	0.958
RPA-class > 4	1.191 (0.898-1.579)	0.225	1.062 (0.731-1.543)	0.751	1.237 (0.789-1.937)	0.354
Incomplete resection	1.498 (1.193-1.881)	0.001	1.126 (0.812-1.560)	0.477	1.889 (1.347-2.647)	<0.001
Simultaneous CT	0.653 (0.488-0.874)	0.004	0.572 (0.398-0.823)	0.003	0.652 (0.381-1.116)	0.119
Fractionation	1.117 (0.865-1.443)	0.396	-	-	-	-
Radiation treatment time	1.010 (0.972-1.049)	0.681	1.008 (0.956-1.064)	0.762	0.997 (0.943-1.055)	0.928
Age	1.021 (1.011-1.032)	<0.001	1.022 (1.008-1.037)	0.003	1.021 (1.005-1.037)	0.009
ECOG state > 1	0.971 (0.791-1.310)	0.846	0.945 (0.631-1.415)	0.785	0.985 (0.619-1.569)	0.950
RPA-class > 4	1.194 (0.901-1.583)	0.218	1.070 (0.736-1.554)	0.749	1.236 (0.790-1.936)	0.354
Incomplete resection	1.510 (1.201-1.899)	<0.001	1.076 (0.779-1.485)	0.435	1.890 (1.350-2.644)	<0.001
Simultaneous CT	0.663 (0.496-0.884)	0.005	0.579 (0.402-0.832)	0.003	0.652 (0.383-1.110)	0.115
Fractionation	1.158 (0.866-1.548)	0.324	-	-	-	-
Overall duration of treatment	1.001 (0.992-1.010)	0.847	1.000 (0.986-1.014)	0.960	1.000 (0.987-1.013)	0.999
Age	1.021 (1.010-1.032)	<0.001	1.022 (1.007-1.036)	0.003	1.021 (1.005-1.037)	0.009
ECOG state > 1	0.969 (0.714-1.314)	0.838	0.934 (0.619-1.411)	0.747	0.986 (0.616-1.581)	0.955
RPA-class > 4	1.191 (0.898-1.579)	0.225	1.062 (0.731-1.542)	0.754	1.236 (0.789-1.937)	0.354
Incomplete resection	1.498 (1.193-1.881)	0.001	1.126 (0.812-1.562)	0.477	1.889 (1.348-2.648)	<0.001
Simultaneous CT	0.652 (0.488-0.871)	0.004	0.570 (0.397-0.819)	0.002	0.652 (0.381-1.116)	0.119
Fractionation	1.123 (0.864-1.458)	0.385	-	-	-	-

The impact of time interval from surgery until initiation of radio- or radiochemotherapy (RCT), radiation treatment time and overall duration of local treatment from surgery to the end of radio- or radiochemotherapy on progression free survival (PFS) is shown

### Waiting time from surgery until initiation of radio- or radiochemotherapy

**Fig. 4:**
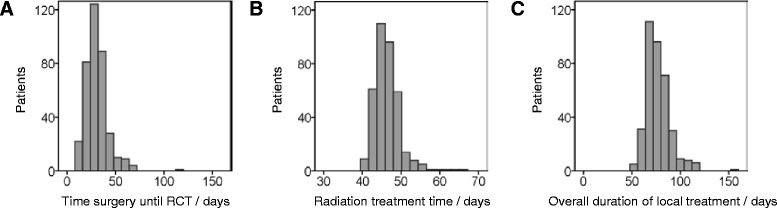
Histograms showing distributions of time intervals investigated. The histograms of time interval from surgery until initiation of radio- or radiochemotherapy (RCT, **a**), of radiation treatment time (**b**) and of overall duration of local treatment from surgery to the end of radio- or radiochemotherapy (**c**) are shown

The time interval (TI) between surgical resection and RCT initiation ranged from 11 to 112 days with a median of 27 days (Fig. 4a). There was no impact of this time interval on OS and PFS both in univariate (Table 2, Fig. 2b) and multivariate analysis (Tables 3 and 4).

The lack of an impact of the time interval between surgery and onset of RCT was not completely unexpected as there are contradictory clinical studies pointing into both directions [13]. There are some studies indicating better outcome after shorter intervals as one would naturally expect from a radiobiological point of view and the knowledge coming from other tumour entities (Burnet, Jena et al. 2006). Some of these exclusively retrospective studies report time interval cut offs distinguishing prognostic groups, e.g. by 37 or 42 days [18, 19]. More precisely, authors report effect sizes in the dimension of survival reduction by 10 weeks per 4 week delay or by 11 weeks per 6 week delay. The same studies quantify an increased risk of death by 2 % for each day of delay or by 8.9 % for each week of delay until the start of radiotherapy [20, 21, 18, 19, 22]. Contradictory to these data, there are several retrospective datasets showing no effect of the time interval on patient outcome [23–26]. More important, prospective clinical data support this missing effect at least for waiting times up to 6 weeks. The largest of all studies mentioned here, compiling prospective data of 2855 patients from 16 RTOG studies, has shown improved outcome for those patients with the longest time until initiation of adjuvant treatment [6]. These unexpected results are not undisputable as patients with better prognostic factors tended to have longer time intervals. Moreover, time interval was not looked upon continuously but considered as an ordinal variable with grouping of patients per week of delay. However, a currently published analysis of 198 patients treated within 4 prospective trials in the temozolomide era confirmed prolonged OS and PFS after short delay with start of postoperative radiochemotherapy at 30–34 days, after adjusting for prognostic factors in multivariate analysis [27]. But on the other hand there is good evidence supporting our results concerning the missing impact of the time interval before adjuvant treatment: Another study aggregating large prospective multicentre data of patients treated within the EORTC-NCIC trial in France did not indicate this time interval affecting patient outcome [24]. One limitation in the interpretation of all prospective clinical trial data is the restriction of the interval to maxima such as 6 weeks according to the study protocols. Thus, these prospective data provide better evidence than the above mentioned retrospective analyses, but they only allow the conclusion that waiting times of up to 6 weeks are safe and maybe somewhat beneficial compared to very short time intervals. Nevertheless, our analysis also does not allow conclusions regarding the impact of very long waiting times, as radiooncologists are aware of a potential negative impact of delays and thus avoid longer time intervals. In our analysis, 10.6 % of all patients had waiting times longer than 6 weeks and 3.5 % longer than 8 weeks. We acknowledge that our study has some drawbacks due to the retrospective nature of our study. However, in contrast to most of the published retrospective studies, our cohort is of appreciable size and is also less heterogeneous than most of the studies, which included different dose levels and sometimes also histologies other than glioblastoma like grade 3 tumours.

### Treatment time of radio- or radiochemotherapy

Radiation treatment time (RTT) ranged from 40 to 71 days with a median of 45 days (Fig. 4b). No effect of varying RTT on both survival endpoints was evident in the whole cohort (Fig. 2c). Main reasons for minimal prolongations were machine breakdowns, patient related factors and public holidays. In many patients, shorter interruptions have been compensated for, e.g. by applying a second radiation fraction on some treatment days, thus keeping the radiation treatment time at 6–7 weeks. Compensation was usually not possible if longer prolongations occurred e.g. due to serious health problems of the patients, such as pulmonary embolism, requiring intensive-care medicine which was the case in the minority of patients. Another issue is that the fractionation schedule using 1.8 Gy per fraction results in a prolongation of 3 days compared to the schedule with 2.0 Gy per fraction. Hence, we looked for differences between and within the two fractionation schemes. The duration varied considerably between 43–71 days and a median of 48 days for 33 × 1.8 Gy versus 40–64 days and a median of 44 days for 30 × 2.0 Gy, but there was no correlation with survival. Furthermore, fractionation scheme was not a significant parameter in univariate (Table 2) or multivariate analysis (Tables 3 and 4).

The data presented here are to our knowledge the first explicitly analysing the impact of treatment time of radio- or radiochemotherapy using homogeneous radiotherapy fractionation schedules on treatment outcome of GBM patients. The missing impact of RTT on outcome is in line with a recently published mathematical modeling study estimating a set of radiobiological parameters from clinical trial outcomes in GBM. The exploration of 559 and validation of 104 patients revealed a tumour doubling time of 37 (range 29–46) days and indicated a long kick-off time for accelerated repopulation as well as only moderate repopulation kinetics in GBM in general. Therefore, the authors concluded independence of the duration of overall treatment [28]. Nevertheless, analysis in the latter publication included different fractionation schemes, as it is also the case in various other studies investigating hypofractionation, so that conclusions concerning time factor cannot be drawn because of combined effects [29]. In contrast to our results and those by Pedicini et al. there are in vitro data showing repopulation in GBM cell lines [30]. Therefore it is possible that strong variations of treatment time could have an effect on survival after all. From our patient cohort, we cannot draw conclusions based on such very long radiation treatment times, because only 8.9 % of all patients had overall treatment times longer than 50 days and 1.1 % longer than 60 days (Fig. 4b). As mentioned above, the policy in our department is to compensate unintended treatment interruptions (in the case of glioblastoma multiforme if they are longer than 3 days) by application of treatment fractions at weekends or second fractions at other treatment days [31, 11]. This is a limitation of the interpretation of our study concerning a missing time factor. The same limitation is also true for prospective clinical trials, as study protocols usually do not allow long prolongations. As the vast majority of patients in our study were treated within radiation treatment times of up to 50 days, a lack of the impact of radiation treatment time on patient outcome can only be concluded for RTT’s up to 50 days. Evidence on the impact of very long radiation treatment times (>50 days) on patient outcome is also not expected in future, because compensation of treatment interruptions is widely implemented in most centres worldwide. Mainly due to ethical reasons, this question cannot be addressed in prospective clinical trials.

### Overall duration of local treatment time from surgery to the end of radio- or radiochemotherapy

Overall duration of local treatment time (ODT) is composed of the interval between surgery and onset of RCT (waiting time TI) and of radiotherapy treatment time (RTT). Both factors were already discussed in detail above. As there was no impact of both TI and RTT, no significant difference could be shown between the groups with short versus long ODT (51–154 days, median 74 days, Fig. 4c) both in the univariate (Table 2, Fig. 2d) and in the multivariate analysis for the whole cohort as well as within the different fractionation schemes (Tables 3 and 4). Supplementary, for all periods of time analysed in this study, results obtained with time and age as continuous variables could be clearly verified by the alternative approach of splitting the cohort by the median of the considered variables both in univariate and multivariate analyses. Results were similarly conclusive for both OS and PFS. In addition to this, we could not show any impact on outcome of all the time variables evaluated (IT, RTT, ODT) neither in the patients with, nor those without simultaneous chemotherapy.

As we could not find a time factor in this analysis, other radiobiological mechanisms than repopulation seem to be more important for aggressiveness and treatment resistance in GBM. Various hypotheses are under investigation. For example, clinical trials are attempting to target intrinsic radioresistance of GBM by treatment intensification using novel combined treatments and selective radiation dose escalation [32]. Another approach is relating to cancer stem cell density and niches, which are also subject of intense research [33, 34].

## Conclusion and outlook

No time factor, neither of waiting time nor of radiotherapy treatment time could be demonstrated for the treatment of GBM in this large contemporary single-centre cohort. Although we recognize that results of retrospective studies harbour important caveats, our data viewed in conjunction with the current literature indicate that short delays of postoperative RCT up to 6–7 weeks, e.g. for screening of a patient for a clinical trial, may be uncritical. Potential delay of treatment start by such procedures is additionally justified by the aim to improve the poor outcome in GBM which will likely require individualization of allocation to new treatment strategies based on good predictive biomarkers.

To the best of our knowledge, these are the first study results indicating absence of any impact of radiotherapy treatment time on clinical outcome of GBM patients. Furthermore, our data suggest that prolongation of radiotherapy treatment time up to 50 days has no impact on overall survival or progression free survival. Conclusions on longer treatment times cannot be drawn based on our data and warrant further investigation.

The missing impact of a time factor in GBM will be validated in a multicentric approach within the Radiation Oncology group of the German Cancer Consortium (DKTK-ROG). To further characterize the cohort presented here, imaging features and elaborate molecular investigations of the paraffin embedded tumour tissue stored in our hospital are planned.

## Abbreviations

CT: Chemotherapy
GBM: Glioblastoma multiforme
ODT: Overall duration of local treatment
RCT: Radio(chemo)therapy
RTT: Radiation treatment time
RPA: Recursive partitioning analysis
RTOG: Radiation Therapy Oncology Group
TI: Time interval from surgery until initiation of radio- or radiochemotherapy
